# Eczema

**Published:** 1902-09

**Authors:** 


ECZEMA. With analysis of eight hundred cases of the disease. By L. Duncan Bulkley, A. M., M. D., Physician to the New York Skin and Cancer Hospital, etc. Third edition of Eczema and Its Management. Entirely rewritten. Pages, 368. Price, $2.00. New York: G. P. Putnam's Sons. 1901.

When it is remembered that one out of every three cases of skin diseases is eczema, it is easy to understand why the author has prepared a work on that subject alone. The author has had a long experience in teaching this subject, and he has made it clear to the average practitioner in the present little volume. This book belongs to the "Students' Manual Series on Diseases of the Skin," but it is quite appropriate to the use of the general practitioner.

